# Using polygenic scores for identifying individuals at increased risk of substance use disorders in clinical and population samples

**DOI:** 10.1038/s41398-020-00865-8

**Published:** 2020-06-18

**Authors:** Peter B. Barr, Albert Ksinan, Jinni Su, Emma C. Johnson, Jacquelyn L. Meyers, Leah Wetherill, Antti Latvala, Fazil Aliev, Grace Chan, Samuel Kuperman, John Nurnberger, Chella Kamarajan, Andrey Anokhin, Arpana Agrawal, Richard J. Rose, Howard J. Edenberg, Marc Schuckit, Jaakko Kaprio, Danielle M. Dick

**Affiliations:** 1 Department of Psychology, Virginia Commonwealth University, Richmond, VA USA; 2 Department of Health Behavior and Policy, Virginia Commonwealth University, Richmond, VA USA; 3 Department of Psychology, Arizona State University, Tempe, AZ USA; 4 Department of Psychiatry, School of Medicine, Washington University in St. Louis, St Louis, MO USA; 5 Department of Psychiatry & Behavioral Sciences, State University of New York Downstate Medical Center, Brooklyn, NY USA; 6 Department of Medical and Molecular Genetics, School of Medicine, Indiana University, Indianapolis, IN USA; 7 Institute for Molecular Medicine Finland, University of Helsinki, Helsinki, Finland; 8 Institute of Criminology and Legal Policy, University of Helsinki, Helsinki, Finland; 9 Faculty of Business, Karabük University, Karabük, Turkey; 10 Department of Psychiatry, School of Medicine, University of Connecticut, Farmington, CT USA; 11 Department of Psychiatry, Carver College of Medicine, University of Iowa, Iowa City, IA USA; 12 Department of Psychiatry, School of Medicine, Indiana University, Indianapolis, IN USA; 13 Stark Neurosciences Research Institute, School of Medicine, Indiana University, Indianapolis, IN USA; 14 Department of Psychological and Brain Sciences, Indiana University, Bloomington, IN USA; 15 Department of Biochemistry and Molecular Biology, School of Medicine, Indiana University, Indianapolis, IN USA; 16 Department of Psychiatry, School of Medicine, University of California, San Diego, San Diego, CA USA; 17 Department of Public Health, University of Helsinki, Helsinki, Finland; 18 Department of Human and Molecular Genetics, School of Medicine, Virginia Commonwealth University, Richmond, VA USA

**Keywords:** Clinical genetics, Predictive markers

## Abstract

Genome-wide, polygenic risk scores (PRS) have emerged as a useful way to characterize genetic liability. There is growing evidence that PRS may prove useful for early identification of those at increased risk for certain diseases. The current potential of PRS for alcohol use disorders (AUD) remains an open question. Using data from both a population-based sample [the FinnTwin12 (FT12) study] and a high-risk sample [the Collaborative Study on the Genetics of Alcoholism (COGA)], we examined the association between PRSs derived from genome-wide association studies (GWASs) of (1) alcohol dependence/alcohol problems, (2) alcohol consumption, and (3) risky behaviors with AUD and other substance use disorder (SUD) criteria. These PRSs explain ~2.5–3.5% of the variance in AUD (across FT12 and COGA) when all PRSs are included in the same model. Calculations of area under the curve (AUC) show PRS provide only a slight improvement over a model with age, sex, and ancestral principal components as covariates. While individuals in the top 20, 10, and 5% of the PRS distribution had greater odds of having an AUD compared to the lower end of the continuum in both COGA and FT12, the point estimates at each threshold were statistically indistinguishable. Those in the top 5% reported greater levels of licit (alcohol and nicotine) and illicit (cannabis and opioid) SUD criteria. PRSs are associated with risk for SUD in independent samples. However, usefulness for identifying those at increased risk in their current form is modest, at best. Improvement in predictive ability will likely be dependent on increasing the size of well-phenotyped discovery samples.

## Introduction

Alcohol misuse is one of the leading contributors to preventable mortality and morbidity worldwide^1–3^. Identifying individuals at heightened risk for developing alcohol-related problems remains an important goal of medical practitioners. One important risk factor for alcohol misuse is one’s own genetic liability. Twin and family studies indicate that genetic influences on alcohol use disorders (AUD) account for ~50% of the variation in the population^4^. Genome-wide association studies (GWASs) have identified multiple variants associated with AUD^5–7^, alcohol consumption^7,8^, and maximum alcohol intake^9^. Using information from these GWASs, we are now able to aggregate risk across the genome by creating polygenic risk scores (PRS) in independent samples^5,6,8,10^.

Beyond being useful for research purposes, researchers have begun to examine the potential of PRS to predict risk for medical outcomes in clinical settings. PRS for coronary artery disease (CAD), atrial fibrillation (AF), type 2 diabetes (T2D), inflammatory bowel disease (IBS), and breast cancer (BC) have been found to be as predictive of these diseases as well known monogenic mutations^11^, which tend to be rarer, and could lead to improved screening for larger numbers of individual who are at risk. Individuals in the top 5% of the PRS distributions had ~3 fold likelihood of having CAD, AF, T2D, IBS, or BC compared to the bottom 95%. For obesity, individuals in the top PRS decile were on average 13 kg heavier than those in the bottom decile^12^. These studies demonstrate the potential for identifying individuals at heightened risk for various medical conditions using PRS. Given that AUD is a moderately heritable trait and GWAS for alcohol-related phenotypes are beginning to identify numerous variants associated with these outcomes, PRS for alcohol-related outcomes may be also able to identify individuals at heightened risk of developing an AUD.

In the current analysis, we tested PRS in two target samples, a population-based sample and a clinically ascertained sample of families deeply affected by AUD, to evaluate the current state of alcohol-related PRS in relation to AUD and identifying those at heightened risk. We use several discovery samples from large-scale GWAS to create three PRS: a meta-analysis of two GWASs on alcohol-related problems^5,6^, a recent large-scale GWAS of alcohol consumption^8^, and a GWAS for risky behaviors, including alcohol use^13^. We chose to test PRS based on multiple alcohol-related GWAS because multiple lines of evidence indicate alcohol consumption and dependence have only partially shared genetic etiology^5,6,14,15^. Additionally, we include a PRS for general risk behavior as there is robust evidence that the genetic risk for alcohol and other substance use disorders is shared with other disorders and behaviors related to reduced inhibitory control^16–18^. Similar to recent work for specific medical conditions^11^, we compare the upper end of the PRS distribution at various thresholds (top 20, 10, and 5%) to examine whether focusing on these upper parts of the distribution provide additional information in identifying those at increased risk of developing an AUD. We acknowledge the exploratory nature of these analyses and the arbitrary nature of our thresholds in the absence of well-defined clinical risk scores, such as those for medical conditions like hypertension. Finally, we test the association of these PRSs with other substance use disorders (including nicotine and illicit substance use disorders), based on the robust finding that substance use disorders share an underlying genetic architecture, with the majority of the heritability shared across substances^16–18^.

## Methods

### Samples

The FinnTwin12 Study (FT12) is a population-based study of Finnish twins born 1983–1987 identified through Finland’s Central Population Registry. A total of 2705 families (87% of all identified) returned the initial family questionnaire late in the year in which twins reached age 11. Twins were invited to participate in follow-up surveys when they were ages 14, 17, and approximately 22 (during young adulthood). An intensively studies sample was selected as 1035 families, among whom 1854 twins were interviewed at age 14. The interviewed twins were invited as young adults to complete the Semi-Structured Assessment for the Genetics of Alcoholism (SSAGA)^19,20^ interview (*n* = 1,347) and provide DNA samples (see Kaprio 2013 for a full description). The current analysis uses data from the young adult wave (mean age = 21.9; range 20–26), which included retrospective lifetime diagnoses.

The Collaborative Study on the Genetics of Alcoholism (COGA) is a sample of high-risk families ascertained through adult probands in treatment for AUD and a smaller set of comparison families from the same communities. In the first 10 years, probands along with all willing first-degree relatives were assessed; recruitment was extended to include additional relatives. Data collection included the SSAGA^19^, neurophysiological and neuropsychological protocols, and collection of blood for DNA. In 2004, COGA began a prospective study of adolescents and young adults, targeting assessment of youth aged 12–22 from COGA families where at least one parent had been interviewed. These young participants were re-assessed every two years. The sample is racially/ethnically diverse (60.6% non-Hispanic White, 24.9% Black, 11.1% Hispanic, and 3.4% other). Most (84%) have GWAS data. A full description of the COGA sample is available elsewhere^21–23^. For the present study, we only focused on COGA participants of empirically assigned (as verified from GWAS data) European ancestry (*n* = 7599) because each of the discovery GWAS samples were primarily of European ancestry. Ancestral mismatch between discovery and target samples can lead to bias in the performance of polygenic scores^24^.

### Measures

#### Alcohol use disorder (AUD)

We used SSAGA interviews to construct lifetime criteria counts of DSM-5 AUD^25^ in each sample. Because individuals in COGA are potentially interviewed multiple times, we used the highest criteria count ever reported by each subject. In FT12, lifetime criteria counts were measured at the young adult interview. In addition to criteria counts, we created AUD thresholds for those who met criteria for mild (2+ criteria), moderate (4+ criteria), or severe (6+ criteria) AUD^25^ without clustering. In both FT12 and COGA, individuals who had never initiated alcohol use were coded as missing.

#### Other substance use disorders (SUD)

We constructed lifetime criteria counts of cannabis, cocaine, and opioid use disorders based on DSM-5 criteria. We measured nicotine dependence criteria using the Fagerstrom Test for Nicotine Dependence (FTND), which assesses six criteria and has values ranging from 0 to 10 in both COGA and FT12. Because many illicit SUDs were not measured or rare in the FT12 data, we limit analyses of illicit SUD to COGA. Like AUD, these criteria counts represent the maximum reported for each respondent across the course of the study. Criteria counts for each substance were limited to those who indicated ever using the corresponding substance. In the case of FTND, this is limited to those who report smoking 100+ cigarettes in their lifetime.

#### Polygenic scores (PRS)

We created PRS derived from publicly available large-scale GWASs. Information on genotyping and quality control is available in the Supplementary information (Section 1). We created PRS using a Bayesian regression and continuous shrinkage method (PRS-CS)^26^. PRS-CS uses LD information from an external reference panel (1000 Genomes Phase III European subsample) to estimate the posterior effect sizes for each SNP in a given set of GWAS summary statistics. Both empirical tests and simulations have shown improved predictive power above traditional methods of score construction^26^. For computational purposes, we limited the SNPS for score creation to HapMap3 SNPs that overlapped between the original GWAS summary statistics, the LD reference panel, and the target samples for score creation. We converted PRS to Z-scores for interpretation.

We used four primary discovery GWASs to create three different PRSs. The first was from a recent GWAS of number of alcoholic drinks per week in approximately one million individuals provided by the GWAS & Sequencing Consortium of Alcohol and Nicotine Use (GSCAN)^8^. We obtained GSCAN summary statistics with all Finnish (which included FinnTwin12) and 23andMe (which are not publicly available) cohorts removed (available *N* = 534,683). The PRS for alcohol problems were derived from a meta-analysis of two GWASs: a GWAS on the problem subscale from the Alcohol Use Disorders Identification Test (questions 4–10; AUDIT-P) in 121,604 individuals from the UK Biobank^6^ and the Psychiatric Genomcs Consurtium’s (PGC) GWAS of alcohol dependence (*N* = 46,568)^5^. Both FT12 and COGA were in the initial AD GWAS and we obtained summary statistics with each cohort removed (meta-analysis results available in supplemental info Tables S1, S2 and Figs. S1, S2). Finally, we derived a PRS for risky behaviors from a GWAS of the first prinicipal component of four risky behaviors (drinks per week, ever smoking, propensity for driving over the speed limit, and number of sexual partners) from 315,894 individuals in the UK Biobank (UKB)^13^. While this PRS does include alcohol consumption and smoking, it captures the shared variance between these substance use measures and the other two risky behaviors. These polygenic scores covered the domains of alcohol consumption (GSCAN DPW), alcohol problems (PROB ALC), and general externalizing (RISK PC).

### Analytic strategy

We first identified the predictive power for each PRS in both COGA and FT12 using the change in R^2^ above a baseline model with sex, age of last observation, the first ten ancestral principal components (PCs), genotyping array, and data collection site (these latter two were only included in COGA analyses). We used linear/generalized-linear mixed-effects models with random intercepts to adjust for clustering at the family level and a pseudo-R^2^ for mixed models^27^. In addition to the predictive power of individual PRS, we estimated the conditional effect of all PRS on AUD criteria to examine whether each PRS explained unique variance in AUD criteria. We also calculated the area under the curve (AUC) of the conditional model containing all continuous PRS to estimate sensitivity/specificity^28^. AUC provides an estimate of the probability a randomly selected case has predicted value more extreme than that of a randomly chosen control^29^. An AUC of 0.5 indicates that a classifier does not provide any useful information in determining cases from controls (see supplemental information Section 3). We next divided PRSs at several thresholds (80th, 90th, and 95th percentiles) to examine whether there was a non-linear increase in risk of AUD (using symptom severity thresholds of AUD) across the PRS continuum. Finally, we compared mean values of other substance use outcomes for the top 5% in each PRS to those in the bottom 95%. We selected this threshold based in the increased prevalence of AUD in those in the top 5% of the PRS distributions (see Fig. S3). All code is available upon request from the corresponding author.

## Results

Table 1 presents the descriptive statistics for each of the samples. Each sample has slightly more female than male participants. The mean number of AUD criteria (3.44) in COGA was relatively high, as COGA was primarily ascertained for families with multiple AUD members. COGA participants report a mean of 4.17 for FTND criteria. For other SUD criteria in COGA, there are a substantial number of participants who report non-zero levels of criteria, though criteria counts for cannabis, cocaine, and opioid use disorders are zero-inflated (see Table 1). The mean number of AUD and FTND criteria in the population-based FT12 sample were 1.63 and 2.57, respectively.

**Table 1:** Descriptive Statistics for FT12 and COGA samples.

Sample		*N*	Mean/%	Median	% 0	SD	Min	Max
COGA	Female	7599	52.84%	–	–	–	–	–
	Age	7599	36.94	–	–	14.77	12	91
	DSM-5 AUD criteria	7300	3.44	2	28.79%	3.63	0	11
	DSM-5 CUD criteria	5051	2.37	1	48.19%	3.13	0	11
	DSM-5 CoUD criteria	2404	3.18	0	50.17%	4.13	0	11
	DSM-5 OUD criteria	1663	2.05	0	62.96%	3.51	0	11
	FTND count	3701	4.12	4	14.02%	2.74	0	10
FT12	Female	1251	54.40%	–	–	–	–	–
	Age	1247	21.94	–	–	0.77	21	26
	DSM-5 AUD criteria	1215	1.63	1	34.57%	1.84	0	11
	FTND count	631	2.57	2	21.55%	2.13	0	10

The *N* reflects those who report lifetime ever use of that substance. All criteria counts limited to individuals who had initiated use of that substance. The % 0 represents the percentage of participants who have initiated use and have no reported criteria.

*AUD* alcohol use disorder, *CUD* cannabis use disorder, *CoUD* cocaine use disorder, *OUD* opioid use disorder, *FTND* Fagerstrom test for nicotine dependence (limited to those who report ever smoking 100 cigarettes).

### Predictive power of PRS

Across each sample and PRS, we observed significant associations between PRS and AUD criteria, even after correcting for a false discovery rate (FDR)^30^ of 5%. In COGA the GSCAN DPW PRS was most strongly associated with AUD criteria (ΔR^2^ = 0.017), followed closely by the RISK PC PRS (ΔR^2^ = 0.016), and lastly the PROB ALC PRS (ΔR^2^ = 0.012). We see a similar pattern in FT12, where the GSCAN DPW PRS was the strongest association (ΔR^2^ = 0.030), the RISK PC PRS was slightly weaker (ΔR^2^ = 0.023), and the PROB ALC PRS performed the worst (ΔR^2^ = 0.001).

**Fig. 1:**
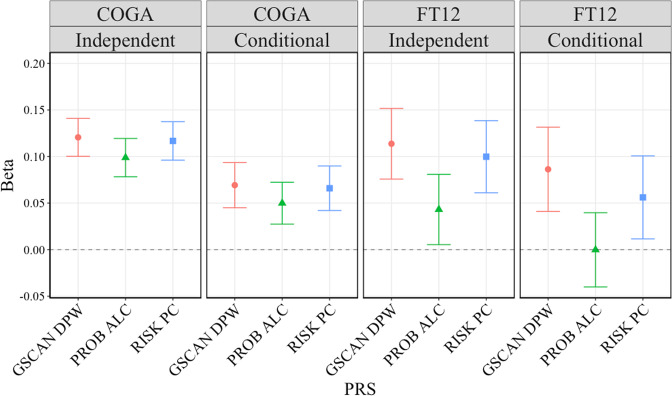
Parameter estimates for PRS in independent and conditional models. Parameter estimates (with 95% confidence intervals), from linear mixed models for alcohol use disorder (AUD) criteria regressed on polygenic risk scores (PRS) for drinks per week (GSCAN DPW), problem alcohol use (PROB ALC), and risky behaviors (RISK PC) in COGA and FT12. Independent = model with only corresponding PRS. Conditional = model with all PRS included. Adjusted for age, sex, first 10 ancestral principal components, genotyping array, and data collection site (only COGA for the latter two). All tests were two-sided.

Next, we determined whether each of these PRS contributed to AUD criteria in a model containing all three, simultaneously. Figure 1 presents the parameter estimates for the independent and conditional effect of each PRS in both COGA and FT12. In the conditional model for COGA, each of the PRSs remains significantly associated with AUD criteria, though the associations are attenuated (conditional model ΔR^2^ = 0.025). In FT12, the PRS for RISK PC and GSCAN DPW remain significant in the conditional model, while the association for PROB ALC PRS is no longer significant (conditional model ΔR^2^ = 0.035). We averaged the three PRS into one composite PRS score in COGA and averaged the RISK PC and GSCAN DPW PRS in FT12 to carry forward in the following analyses.

**Fig. 2:**
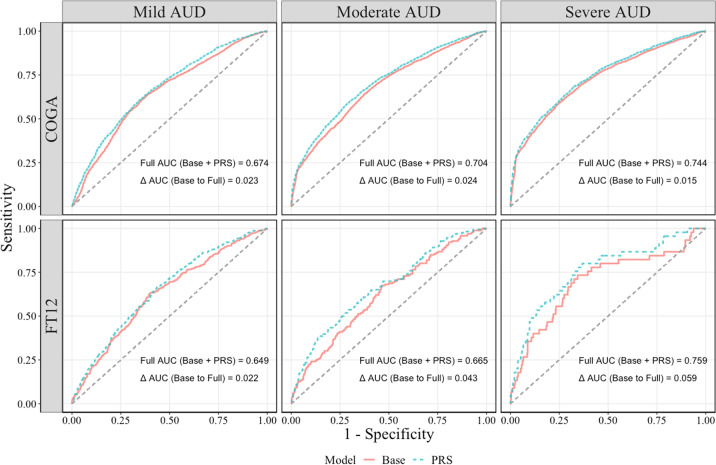
ROC curves for baseline and PRS models. Receiver operating characteristic (ROC) curves for baseline models (covariates only) and polygenic risk score (PRS) models (PRS + covariate) for each level of severity in alcohol use disorder (AUD). Area under the curve (AUC) for the PRS model (Full AUC) and change in in AUC from Base to PRS model (Δ AUC) is presented each cell. AUC provides an estimate of the probability a randomly selected subject with the condition has a test result indicating greater suspicion than that of a randomly chosen subject without the condition^29^. An AUC of 0.5 indicates that a classifier does not provide any useful information in determining cases from controls.

Finally, we assessed the sensitivity/specificity of these combined PRS by calculating the AUC from receiver operating characteristic (ROC) curves, presented in Fig. 2. AUC from the full model (including both continuous PRS and covariates) for each level of AUD severity ranged from 0.67 to 0.74 in COGA and from 0.65 to 0.76 in FT12. Comparing the AUC for the models with and without PRSs, including the PRS only provided a slight increase in AUC.

### Increase in risk across the polygenic continuum

In order to estimate whether individuals at the extreme end of the PRS distribution were at elevated risk of AUD, we compared the risk of AUD between those above and below a given threshold in the distribution. We divided these PRSs at the 80th, 90th, and 95th percentile in each sample and estimated the odds ratio (OR) for AUD in the top portion of the distribution relative to the bottom portion of the distribution (e.g., splitting at the 80th percentile compares the top 20% to the bottom 80%). Table 2 provides the estimates for all of those models. Across each threshold for AUD severity in COGA, we observed a similar pattern where, as expected, those in the upper end of the polygenic distribution had greater odds of meeting criteria for AUD. However, regardless of the threshold, the OR’s at each threshold were roughly equivalent. For example, in the case of severe AUD, when dividing 80th percentile (OR = 1.98; 95% CI = 1.53, 2.57), 90th percentile (OR = 2.02; 95% CI = 1.73, 2.36), or 95th percentile (OR = 1.96; 95% CI = 1.59, 2.40), all of confidence intervals for the point estimates overlap. In FT12, there was a similar pattern. Though some of the point estimates appear to increase as the thresholds become more restrictive, the confidence intervals again overlap.

**Table 2:** Odds ratios for those at extreme end of the PRS continuum.

Sample	Phenotype	Prevalence	Split	*N* Cases	OR	95 % CI Low	95 % CI High
	Mild AUD	57.06%	80%	998	1.96*	1.70	2.26
COGA	Mild AUD	57.06%	90%	501	1.81*	1.49	2.19
	Mild AUD	57.06%	95%	258	1.89*	1.45	2.47
	Moderate AUD	37.44%	80%	738	2.07*	1.79	2.38
COGA	Moderate AUD	37.44%	90%	383	1.94*	1.60	2.34
	Moderate AUD	37.44%	95%	201	1.98*	1.53	2.57
	Severe AUD	25.89%	80%	534	2.02*	1.73	2.36
COGA	Severe AUD	25.89%	90%	285	1.96*	1.59	2.40
	Severe AUD	25.89%	95%	146	1.81*	1.38	2.39
	Mild AUD	41.98%	80%	123	1.78*	1.21	2.61
FT12	Mild AUD	41.98%	90%	68	2.35*	1.41	3.93
	Mild AUD	41.98%	95%	32	1.94	0.97	3.88
	Moderate AUD	13.91%	80%	55	2.85*	1.72	4.74
FT12	Moderate AUD	13.91%	90%	32	3.50*	1.85	6.64
	Moderate AUD	13.91%	95%	15	3.14*	1.33	7.42
	Severe AUD	3.79%	80%	16	2.84*	1.37	5.87
FT12	Severe AUD	3.79%	90%	12	4.41*	2.04	9.54
	Severe AUD	3.79%	95%	5	2.98	0.96	9.30

All models control for sex, age at last interview, and first 10 principal components. Models for COGA also included data collection site and genotyping array. *N* Cases = number of individuals who meet criteria for a given level of AUD and are in the top portion of the split.

*p < 0.05 (two-sided) after correcting for 5% false discovery rate (FDR).

### Examining the substance use phenome of the extreme end of the polygenic risk continuum

**Fig. 3:**
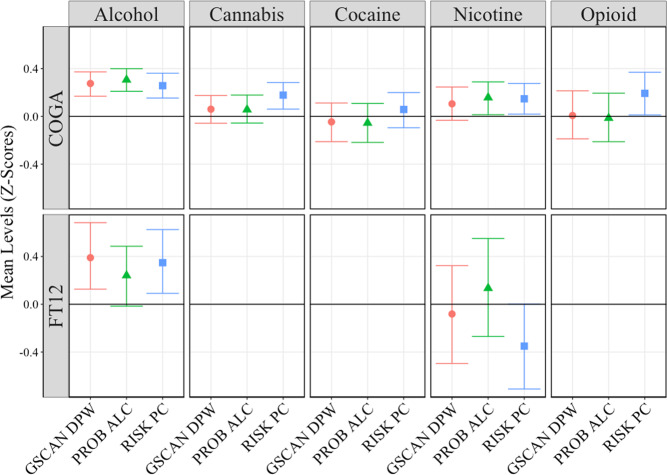
Top 5% of PRS Continuum. Mean levels of substance use disorder (SUD) criteria for alcohol, cannabis, cocaine, nicotine, and opioid use disorders for top 5% of each polygenic risk score (PRS) compared to the bottom 95%. Black bar represents mean of bottom 95% of each sample. 95% confidence intervals estimated using 1000 bootstrap resampling.

We compared the likelihood of substance-related outcomes in individuals in the top 5% of each of the PRS in COGA and FT12 (adjusted for covariates). Figure 3 presents the mean lifetime criteria endorsed for a variety of substance use disorders (alcohol, cannabis, cocaine, nicotine, and opioid) for individuals in the top 5% for each PRS relative to the bottom 95% of each PRS. In COGA, individuals in the top 5% of the PROB ALC, RISK PC, and/or GSCAN DPW PRS had significantly higher levels of alcohol (0.25–0.31 SD), while individuals in the top 5% of the PROB ALC and RISK PC had higher mean nicotine criteria (0.10–0.16 SD) than those in the bottom 95% of the PRS distribution. Those in the top 5% of the RISK PC PRS also endorsed a higher number of criteria for cannabis use disorder (0.14 SD) and opioid use disorder (0.19 SD). In FT12, those in the top 5% in the top 5% of the RISK PC and GSCAN DPW PRS had significantly higher levels of AUD criteria (0.25–0.31 SD) but did not differ on FTND criteria.

Overall, individuals in the top 5% of any PRS report greater levels of any substance, though being in the top 5% of the RISK PC PRS is associated with the most other substances. These PRS are modestly correlated with one another in both COGA (*r*_RISK PC* PROB ALC_ = 0.34; *r*_GSCAN DPW*RISK PC_ = 0.49; *r*_GSCAN DPW* PROB ALC_ = 0.40) and FT12 (*r*_RISK PC*PROB ALC_ = 0.25; *r*_GSCAN DPW*RISK PC_ = 0.50; *r*_GSCAN DPW* PROB ALC_ = 0.35), but each captures unique information related to the genetics of substance use problems (and other risky behaviors).

## Discussion

Researchers have begun to evaluate the potential for use of PRS (for a variety of medical phenotypes)^11,12^ in clinical settings. In this analysis, we examined the current predictive power and strength of association between several PRSs and a variety of SUDs, with a focus on AUD in both a clinically ascertained and a population-based sample. We were interested in (1) which scores based on available GWASs provided the strongest association with alcohol use disorder, whether these scores explained unique variance in AUD in a conditional model, and how well these scores discriminated between cases and controls; (2) what the risk of AUD was for those at the upper end of the risk continuum compared to the bottom; and 3) the levels of substance use disorder criteria for individuals at the top 5% of the polygenic score continuum compared to remaining 95%.

In terms of which polygenic scores were the most predictive, we considered three scores: one based on problematic alcohol use (PROB ALC), one based on alcohol consumption (GSCAN DPW), and one based on general risky behaviors (RISK PC), as twin and family studies have shown alcohol and other risk behaviors to be genetically correlated traits^6,14–18^. In both samples, the GSCAN DPW PRS was the most strongly associated, followed closely by the RISK PC PRS. When we included all of the PRS in one model, all three PRS were associated with AUD criteria in COGA. Only the RISK PC and GSCAN DPW PRS were associated with AUD criteria in FT12. Overall, the unique contributions of each PRS reinforce the notion that the genetics of AUD are multifaceted, comprised of risk for level of consumption, alcohol-related problems, and behavioral disinhibition^31,32^. Evaluating the AUC for the combined PRS revealed the combined effect of PRSs only marginally improved the AUC, similar to recent analyses for coronary artery disease^33^ and ischemic stroke^34^. We ran a series of sensitivity analyses to test whether differences across the samples reflected age differences rather than differences in ascertainment. Restricting COGA to participants under 30 did not fundamentally change the results (see Supplementary information Section 4, Table S3, and Fig. S4). Evaluating the AUC for the combined PRS revealed the combined effect of PRSs only marginally improved the AUC over models with just covariates.

In an exploratory approach, we chose a series of more restrictive thresholds to divide the PRS distribution. The odds of having an AUD were statistically indistinguishable across each of the thresholds in both COGA and FT12. Even though the point estimates increased in some cases, the confidence intervals around these estimates were relatively large and they did not differ significantly. Additionally, there were only a small number of individuals in the severe category in FT12 and we urge caution in interpreting these estimates. Finally, the top 5% of the continuum for each PRS reported elevated rates of other SUD criteria (cannabis, cocaine, opioid, and nicotine use disorders) compared to the bottom 95%. The RISK PC PRS was most associated with higher mean levels of SUD criteria, suggesting that risk for externalizing may be particularly useful in identifying individuals at risk for multiple SUDs.

These initial findings suggest the current PRSs are unlikely to prove useful for SUDs in a clinical setting. Being able to eventually identify those at heightened risk for SUDs may allow for more targeted early intervention and prevention. However, before this is possible, larger discovery GWAS across substance use phenotypes with PRS that explain greater portions of the variance will be necessary. As GWAS sample sizes for SUDs increase, we will likely see increases in effect sizes^35^. Additionally, using multivariate techniques to model the shared genetic architecture across existing SUD GWAS to include both aspects of externalizing and internalizing (e.g. depression, anxiety) may also improve prediction^36,37^. Inclusion of genetic data in a clinical setting will also require that psychiatrists and clinicians receive greater training in genetics and/or that they partner with genetic counselors, so they are both better able to understand what increased genetic risk means and be able convey that information accurately to their patients^38,39^. In addition to clinical utility, we must ensure that regulations and protections surrounding the use of genetic information in clinical settings can adequately protect the rights of individuals who are identified to be “at risk.”

This research has several important limitations. First, all analyses were limited to individuals of European ancestry because the discovery GWASs available were conducted in individuals of primarily European ancestry. It will be important to ascertain sizable samples of subjects with non-European ancestries to properly estimate the predictive utility of PRS in non-European samples. This is especially important for racial-ethnic minorities so that health disparities are not further perpetuated^40^. Second, our use of lifetime diagnoses may obscure the impact of changing genetic influences on the development of AUD across the life course^41,42^. Future work should draw on longitudinal data to examine the ways in which the strength of associations for PRS changes with the age of the target sample. Third, UKB was a large portion of the discovery sample for each of the GWAS used to create PRS. To the degree that UKB is biased, each of the PRS in these analyses will also reflect that bias^43^. Finally, these analyses examined the marginal influence of PRS, independent of environment. Processes of gene-environment interaction (GxE) are well documented in alcohol misuse^44–47^. Incorporating environmental information along with PRS in a methodologically rigorous manner will be an important next step in developing clinically predictive algorithms.

Polygenic scores are becoming better powered and starting to explain non-trivial portions of variance. We examined the current state of PRS for substance use, with a focus on AUD. Each of the PRSs analyzed here were associated with AUD. However, the maximum variance explained by any single score was still small (~2%). Individuals at the top of the PRS continuum had elevated rates of multiple substance use problems, but these differences across the PRS continuum are unlikely to be of broad clinical use in their current state. As GWAS discovery samples become larger and we are better able to model the complex relationship between genotype and phenotype, polygenic scores may eventually be useful in a clinical setting.

## Supplemental information

Supplemental information
